# Noncontact tip-enhanced Raman spectroscopy for nanomaterials and biomedical applications†

**DOI:** 10.1039/c9na00322c

**Published:** 2019-08-19

**Authors:** Dmitry N. Voylov, Vera Bocharova, Nickolay V. Lavrik, Ivan Vlassiouk, Georgios Polizos, Alexei Volodin, Yury M. Shulga, Alexander Kisliuk, Thirumagal Thiyagarajan, Duane D. Miller, Ramesh Narayanan, Bobby G. Sumpter, Alexei P. Sokolov

**Affiliations:** Department of Mechanical Engineering, Tufts University Medford Massachusetts 02155 USA dmitry.voylov@tufts.edu dvoylov@utk.edu; Chemical Sciences Division, Oak Ridge National Laboratory Oak Ridge Tennessee 37831 USA; Center for Nanophase Materials Sciences, Computational Sciences and Engineering Division, Oak Ridge National Laboratory Oak Ridge Tennessee 37831 USA; Energy & Transportation Science Division, Oak Ridge National Laboratory Oak Ridge Tennessee 37831 USA; Institute of Problems of Chemical Physics RAS Chernogolovka Moscow region 142432 Russia; National University of Science and Technology MISIS Moscow 119049 Russia; Department of Medicine, University of Tennessee Health Science Center Memphis Tennessee 38103 USA; Department of Pharmaceutical Sciences, University of Tennessee Health Science Center Memphis Tennessee 38103 USA

## Abstract

Tip-enhanced Raman spectroscopy (TERS) has been established as one the most efficient analytical techniques for probing vibrational states with nanoscale resolution. While TERS may be a source of unique information about chemical structure and interactions, it has a limited use for materials with rough or sticky surfaces. Development of the TERS approach utilizing a non-contact scanning probe microscopy mode can significantly extend the number of applications. Here we demonstrate a proof of the concept and feasibility of a non-contact TERS approach and test it on various materials. Our experiments show that non-contact TERS can provide 10 nm spatial resolution and a Raman signal enhancement factor of 10^5^, making it very promising for chemical imaging of materials with high aspect ratio surface patterns and biomaterials.

## Abstract 2

Demonstration of a proof of the concept and feasibility of a non-contact TERS approach promising for imaging of materials with high aspect ratio surface patterns and biomaterials.

## Graphical abstract

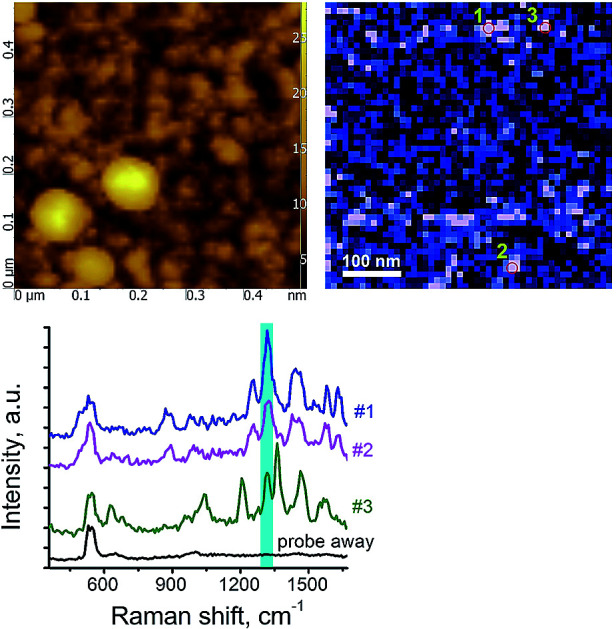

## 1. Introduction

Tip-enhanced Raman spectroscopy (TERS) is a powerful technique for chemical imaging with nanoscale lateral resolution, combining advantages of scanning probe microscopy (SPM) and Raman spectroscopy.^1^ TERS utilizes different kinds of plasmonic structures attached to the SPM tips, providing an enhancement of the electrical field of light in a near proximity to the tip apex. One of the critical aspects of TERS is the type of SPM feedback as well as, type, shape and location of the plasmonic material at the tip apex. While scanning tunneling microscopy based TERS provides the best lateral resolution,^2^ it has significant restrictions for the samples, *e.g.*, it is limited to atomically or molecularly thick layers or conductive materials.^3^ Therefore, the majority of TERS applications employ a contact AFM mode using inverted illumination optical configuration.^4,5^ However, the latter limits the TERS use to only transparent samples on transparent substrates. As a result, many interesting non-transparent materials, and nanostructures are not accessible for TERS measurements with the inverted configuration. Fortunately, there is an alternative approach – the use of side or top illumination geometry. The side illumination geometry allows a much better TERS signal in comparison to the top illumination, because the latter is strongly hindered by the probe shadow that blocks major scattering signal. At the same time, regardless of the optical geometry, the contact TERS technique has several significant disadvantages, including high probability of tip contamination by analyte and tip damage in the case of rough and rigid materials. There are many reports^6–9^ where such resonance techniques as tapping mode and tuning fork were used. Yano *et al.*^10^ and Yu *et al.*^11^ reported development of elegant approaches of the tapping-mode TERS allowed accumulation of the far and near field Raman signals during one measurement. While it required a slight modification of the AFM-Raman system, basic considerations clearly indicate a great potential of the resonant mode TERS in applications for materials with rough or “sticky” surfaces. At the same time, many reports showed that so called true non-contact AMF mode,^12^ at which the resulting tip/sample force is purely attractive, significantly benefits study of topology of such materials.^13–15^ Therefore, utilizing a non-contact resonant AFM mode in TERS measurements has the potential to enable measurements to be less prone to contamination which is critical for many applications including bio- and organic materials, nanostructures, *etc.*

Here we show the capability of non-contact mode TERS using several carbon-based nanomaterials and a protein–drug mixture. We start with a detailed description of the experimental setup, tip preparation and alignment procedure. To test reasonable sensitivity and resolution of non-contact mode TERS we measured multiwall carbon nanotubes and graphene oxide on silicon substrate using 532 nm and 638 nm wavelength lasers. We mapped the TERS signal from graphene on top of a poly (methyl methacrylate) (PMMA) polymer film using 532 nm light. Finally, we tested the non-contact TERS by imaging a protein–drug mixture – ‘sticky’ bio-organic material which has a high tendency for tip contamination and high fluorescence.

## 2. Experimental section

### 2.1 Materials

#### Carbon nanotubes

For the synthesis of carbon nanotubes, a catalyst Fe–Mo/MgO containing 3 wt% of Fe–Mo (molar ratio Fe : Mo = 5 : 1) was used. The procatalyst was prepared by impregnation of a highly dispersed MgO powder with an aqueous solutions mixture of the Fe(NO_3_)_3_ and (NH_4_)_6_Mo_7_O_24_ with subsequent drying in air at 100 °C. The reduction and activation of Fe–Mo catalyst occurred directly during the synthesis of nanotubes. CNT were synthesized in a gas flow reactor by methane pyrolysis from the gas mixture CH_4_ : H_2_ = 3 : 1 at 900 °C and 0.1 MPa for 1 hour. Carbon products of pyrolysis were purified from the carrier and catalyst by ultrasonic treatment in concentrated hydrochloric acid at 80 °C for 3 hours followed by flushing in distilled water and drying in air at 100 °C.

Syntheses of graphene and graphene oxide were described in detail in ref. 16 and 17 respectively.

#### Protein

Androgen Receptor (AR) activation function-1 domain (AF-1) that spans amino acids 101–370 was cloned into bacterial expression vector pGEX 4t.1. The bacterial culture with the cloned plasmid was incubated in a 37C shaker until it attained O.D. 600 nm. The protein expression was induced using IPTG and incubated at 25C for 6 hours. The bacterial cells were pelleted, and protein extracted as described before (Our Cancer Research Paper). The protein was purified using glutathione S transferase (GST) column and further purified using FPLC.

#### UT-155 (Drug)

UT-155 is a selective androgen receptor degrader (SARD) that binds to both the AF-1 domain and the LBD. The molecule antagonizes and degrades the AR and AR splice variants (AR-SVs) at nanomolar or sub-micromolar doses.

### 2.2 Sample preparation

Graphene on Cu substrate was first spin coated with a layer of poly(methyl methacrylate) (PMMA) (495 A4, Microchem) at 2000 rpm for 45 s followed by etching the metal with CE-100 etchant (transene). PMMA/graphene film was washed by 10% HCl solution and DI water and transferred onto a Si wafer.

Graphene oxide and Carbon nanotubes were drop casted from water solution on Si wafer. After 5 minutes the droplets were removed with filter paper. Silicon wafers (TED PELLA, USA) surfaces were preliminary cleaned with 2% KOH/water solution and rinsed with deionized water. After deposition, all samples were dried in vacuum at room temperature for 5 hours.

Stock solution of protein and UT-155 was prepared by mixing 10 ml of protein conc. 12 mg ml^−1^ (buffer exchanged with 20 mM phosphate buffer pH 6.8) with 2.5 ml of drug (100 mM in DMSO). Volume of the mixture was adjusted to 500 ml by adding phosphate buffer (20 mM pH 6.8). The stock was dilute 10×, 100× and 1000× time in phosphate buffer. Resulting solutions were spin coated on the Al mirrors (4000 rpm acceleration 100 rpm in m) for 300 s yielding samples with the reduced surface concentration.

### 2.3 Density functional theory (DFT) calculations

Theoretical calculations in our studies are used to predict electronic structure of drug and amino acids of the corresponding Raman response/spectra interactions with the noble goal of identification of the interaction between the drug and protein. The theoretical approach used is based on the density functional tight binding (DFTB) framework to describe the electronic structure. This is an approximate density functional theory in which only valence electrons are treated quantum mechanically while all core electrons and nuclei are treated a *via* pairwise interatomic repulsive potential *E*_rep_.1
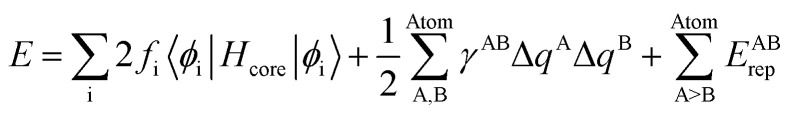
where *f*_i_ is an occupation number (typically 0 or 1) and i runs over all molecular orbitals. The first term describes interaction of valence electrons with core ions (nuclei and core electrons). The second term is responsible for electron–electron interaction. Symbols Δ*q*^A^ and *γ*^AB^ are, respectively, a charge at center A and a chemical hardness' based parameter depending on interatomic distance that describes electron–electrons interactions between centers A and B. The last term describes interaction between core ions obtained from a fit.^18,19^ The DFTB method fills the gap between classical force fields and density functional theory. Importantly for the present project, is that it is about 1000 times computationally cheaper than density functional theory. The main reason for lower computational cost is due to the facts that (a) only valence electrons are considered while core electrons are neglected, (b) a minimal basis set is used (Slater basis), (c) only pair-integrals are used in calculations. A consequence of (a) and (b) is that for a given molecular system all matrices are 5–10 times smaller. As a consequence of (c) the cost of formation of all matrices is significantly lower than in DFT. Overall this savings in computational time allows more facile treatment of realistic size of systems, as is required for the present study. Raman spectra were computed from DFTB optimized structures using the method developed by Witek *et al.*^20^

### 2.4 TERS probes preparation

Before silicon probes metallization, they were oxidized. Oxidation of the SPM probes was carried in pure oxygen at the atmospheric pressure at 1000C for 30 minutes using a rapid thermal processing tool (First Nano, Inc.) Thickness of the oxide grown on satellite silicon chips under these conditions was determined to be approximately 50 nm.

The chips with SPM probes were mounted on a rotating sample holder with rotation axis inclined at approximately 15 degrees with respect to the source-sample line. Metal films were deposited on SPM probes in a vacuum evaporator using metal sources mounted on a carousel and heated by an electron-gun. A stack of 5 nm Cr, 40 nm Ag and 2 nm Al was deposited under vacuum of at least 5 × 10^−6^ Torr without opening the chamber. Deposition rates and effective mass thicknesses of the deposited films were monitored by a quartz crystal microbalance. Deposition rates were approximately 0.1 nm s^−1^ for each of the three metals.

### 2.5 Experimental setup and alignment procedure

**Fig. 1:**
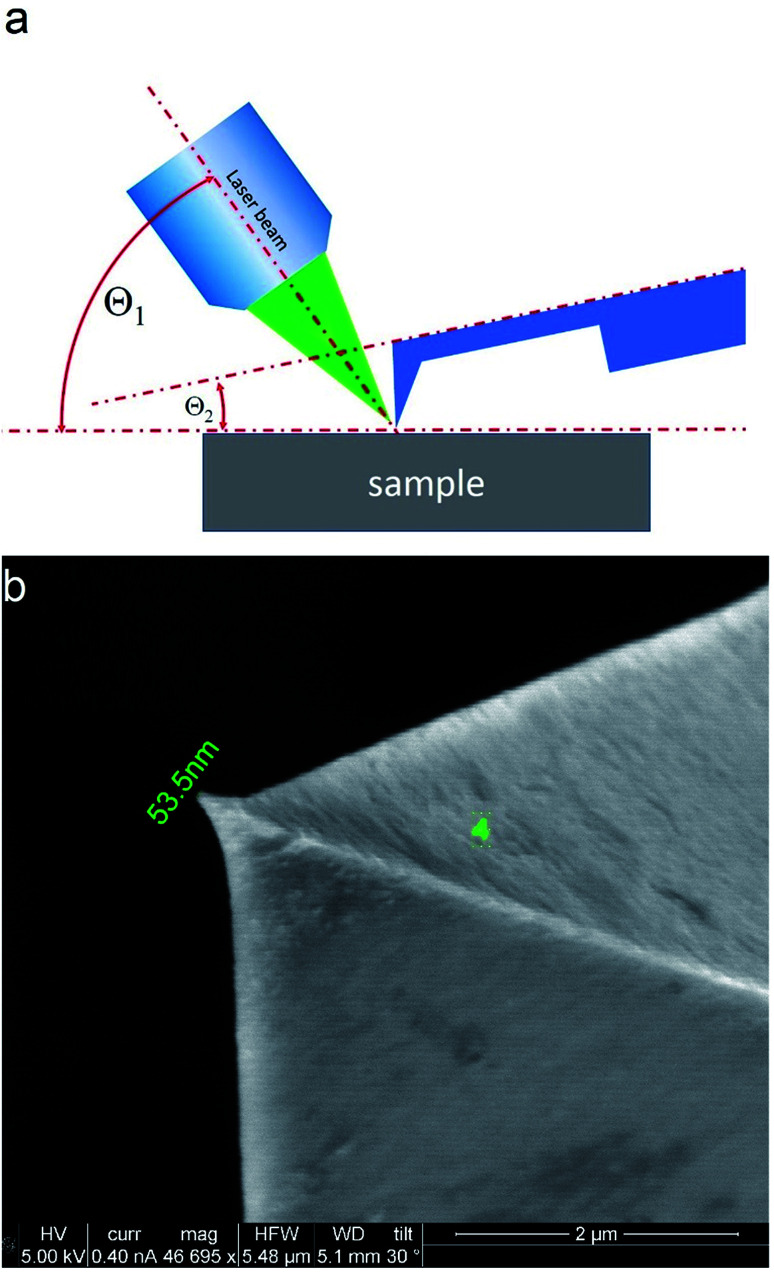
Scheme of the side illumination configuration (a) and SEM image of metallized probe (b). In this configuration the angles between the sample surface and principle axis of the objective *Θ*_1_, and between sample surface and cantilever plane *Θ*_2_ were fixed and equal to approximately *Θ*_1_ ≈ 35°, and *Θ*_2_ ≈ 12°.

TERS measurements were performed using AIST-NT (Novato, USA) AFM-Raman system based on an OmegaScope scanning probe microscope with side and top illumination configurations. All results presented in this work were obtained with the side illumination geometry (Fig. 1 a) using a 100× Mitutoyo Plan Apo SL Infinity Corrected Objective (working distance 13 mm, and numerical aperture 0.55), and a probing wavelength *λ* = 532 nm and *λ* = 638 nm. For metallization, OTESPA-R3 (Bruker) AFM probes with resonance frequency *f* ∼300 kHz, and spring constant 26 N m^−1^ were chosen. The main advantage of these probes is top visibility of the tip apex, high spring constant, stability and high-quality imaging in non-contact and tapping modes. We found, that working amplitudes for reliable TERS signal with this type of AFM probes were in a range between 5 and 15 nm and depend significantly on the surface roughness and physicochemical properties of studied materials. All TERS measurements were done using AFM noncontact mode (see ESI† for more details) at a laser power density of ∼1.53–3.2 × 10^7^ W m^−2^ (laser power 12–25 μW). Higher laser power (above 120 μW) led to abrupt disappearance of the TERS signal, which we believe is related to a melting and destruction of the silver plasmonic structure.

**Fig. 2:**
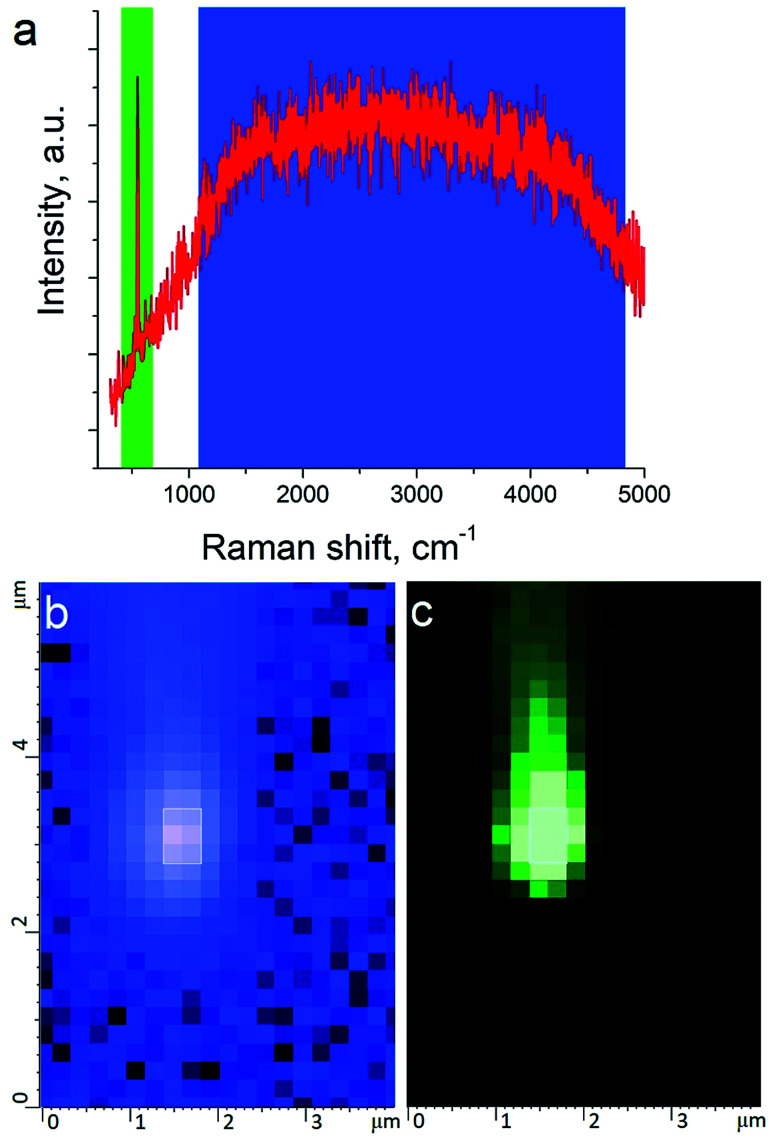
Raman spectrum and map of the TERS tip. (a) Raman spectrum of the tip where blue and green color indicate fluorescence from plasmonic structure and the main silicon peak at 520 cm^−1^ respectively. (b) Map of intensity of fluorescence shown in (a) with blue color. (c) Map of Raman intensity of silicon peak at 520 cm^−1^ shown in (a) with green color.

The tip metallization procedure included deposition of adhesion Cr layer ∼5 nm, Ag layer ∼50 nm, and Al layer ∼2 nm to protect the tips from degradation at ambient conditions, as was described in several reports.^21,22^ Scanning Electron Microscopy (SEM) image of the probe after metallization is shown in the Fig. 1b. Before metallization, some of the tips were oxidized according to procedure similar to the one described by Hayazawa *et al.*^23^ We found, that indeed, oxidation of the tips before metal deposition positively influenced reproducibility of tip preparation and TERS signal enhancement (see ESI† for more details). The alignment procedure started with a manual focusing of a laser spot on the tip end using adjustment screws manipulating the position of the tip with respect to the laser position, visually monitoring the progress with an optical microscope and video camera (see ESI† for more details). After the best possible focus of the tip end was achieved and aligned with the laser spot, the Raman signal from the tip was mapped in vertical and horizontal directions with the objective mounted on piezo drivers (Fig. 2a and b). The latter procedure aims to find the so called “hot spot” – location of the highest fluorescence signal on the tip end (Fig. 2c). Additional scanning of the signal dependence on objective focus was performed if necessary. Landing was done with the setpoint amplitude set in the range from 92 to 97% of free amplitude of the tip (retracted from the surface) and adjusted afterwards. In order to control and maintain a non-contact regime both oscillation amplitude and phase were used as feedback parameters. The final adjustment of the setpoint was based on the distance dependence of probe amplitude, with the main criteria to avoid hysteresis in distance dependence, and to have a stable amplitude and phase behaviors. Here, by stability we mean an optimal setpoint which is as close to free amplitude as possible to allow performing topology mapping without “losing” the contact between the tip and surface. It is always a compromise between a stable control of scanning performance through the microscope feedback system and smaller interaction forces between tip and surface. Therefore, the working setpoint and cantilever amplitude depend strongly on the probe-material interaction. All results presented here were measured at least twice to confirm reproducibility.

## 3. Results and discussion

### 3.1 Carbon nanotubes on silicon wafer

**Fig. 3:**
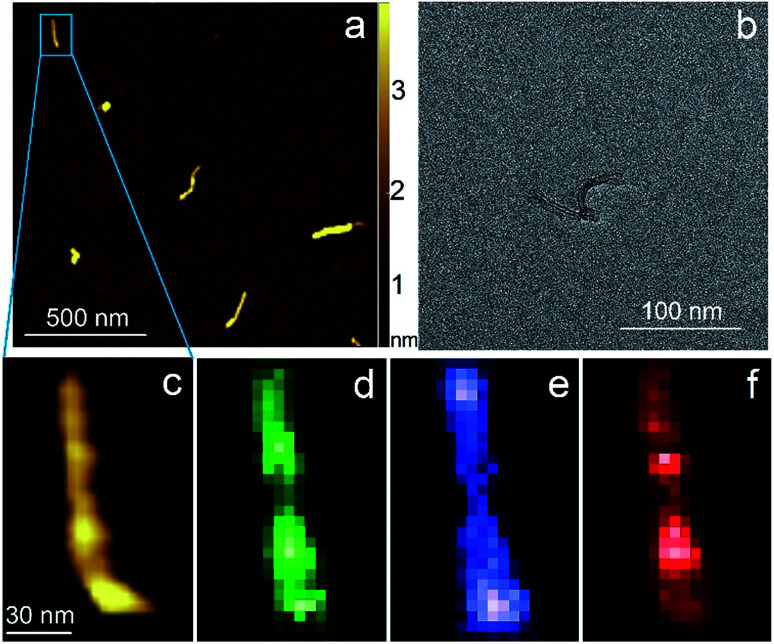
AFM (a) (c) and TEM (b) images of CNTs. TERS mapping of the G band (d), D band (e), 2D band (f) intensity maps of the nanotube shown on (a) and (c).

**Fig. 4:**
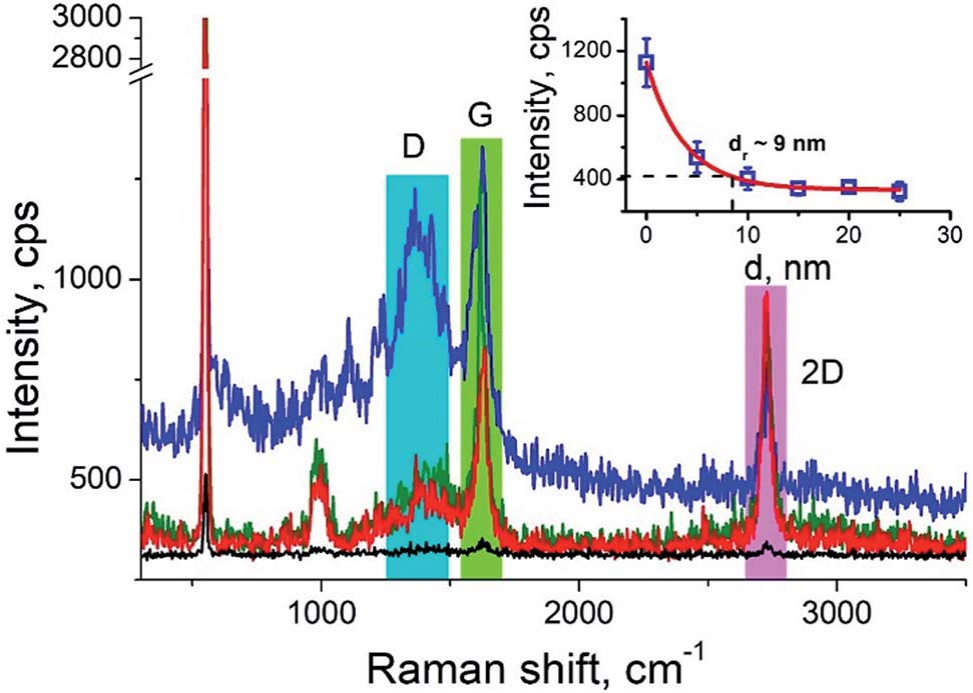
Characteristic TER spectra of CNTs with from three different positions: at the edge (blue), in center (green) and (red), and when tip is withdrawn on 15 nm (black). The inset shows the plot of G band intensity *versus* distance from the nanotube edge. Blue, green and red bars show integration limits for calculation of area under the peaks.

Carbon nanotubes (CNTs) are good test samples for TERS experiments because they give a strong Raman signal and their small diameter enables reliable estimates of the TERS spatial resolution and electrical field enhancement.^4^ For the measurements we used bundled multiwall carbon nanotubes chemically cut before measurements. CNTs were deposited on a silicon wafer (TED PELLA, USA) from aqueous solution, which was intensively sonicated for 30 minutes before deposition. The average length of the CNTs was estimated from AFM and Transmission Electron Microscopy (TEM) measurements (Fig. 3a and b) and it was around 80 nm with a broad distribution. We found the longest CNTs were up to 350 nm in length, while the shortest about several nanometers. The typical images occurred with AFM and TEM are shown in the Fig. 3a and b. The TERS mapping and spectra are shown in the Fig. 3d–f. Typical Raman spectra of carbon nanotubes and graphene consist of a first-order Raman scattering process known as a G band (around 1600 cm^−1^), 2D (or G′) band (∼2700 cm^−1^) originated from a two-phonon Raman scattering mechanism, and D band (∼1350 cm^−1^) associated with defect structure. Calculations show that the intensity (here and after intensity means area of the peak) of G band decreases ∼*e* (∼2.7) times at a lateral distance of about 9 nm (Fig. 4 inset), which may be considered as a spatial resolution for the TERS experiment. For this measurement a 532 nm wavelength laser was used with a power density 3.2 × 10^7^ W m^−2^ (25 μW). A signal accumulation time (or exposure time) was set to 0.5 s per point. The amplitude of tip oscillations was estimated as ∼5 nm. The setpoint was set as 95% from free oscillation amplitude (when the tip is withdrawn).

The electromagnetic enhancement factor is an important parameter characterizing enhancement of the Raman signal from the area under tip. It may be estimated using: 2
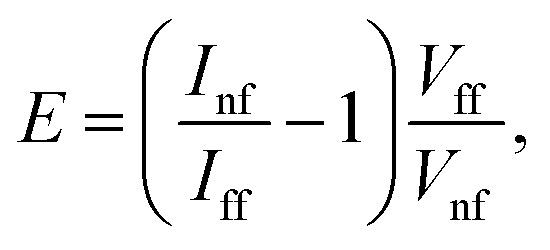
where *I*_nf_, *I*_ff_ – near (tip approached) and far (tip withdrawn) field intensities of the Raman signal; *V*_nf_ and *V*_ff_ – the volumes probed by the far- and near-fields, respectively.^24^ The near field Raman intensity corresponds to the intensity measured from the volume *V*_nf_ = (*R*_tip_/2)^2^π*d*_nf_, when the tip is landed and located in a proximity to the sample surface. Here *R*_tip_ is the curvature radius of the tip, *d*_nf_ is the thickness of the sample from which the TERS signal *I*_nf_ is accumulated. Accordingly, when the tip is retracted, the Raman intensity corresponds to the far field intensity *I*_nf_, which is collected from the volume *V*_ff_ = *R*^2^_focus_π*d*_ff_. Here *R*_focus_ is the focal radius and *d*_ff_ is the thickness of the sample from which the far field Raman signal *I*_nf_ is accumulated. In the case of reasonably thin films, the ratio of volumes may be substituted by areas:^25^3
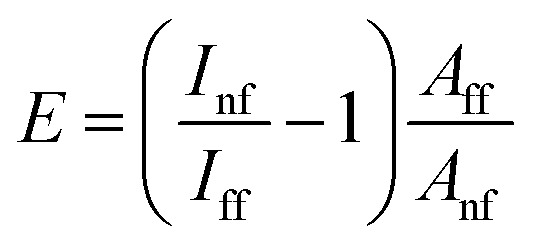


For calculations of the enhancement factor at TERS measurements of CNTs, when individual nanotubes are well separated from each other and do not cover a significant area of the substrate surface, we may simplify eqn (3) by taking the ratio of the nanotube length under the tip *L*_nf_ to the total average length of CNT in the area within the focal radius *L*_ff_:4
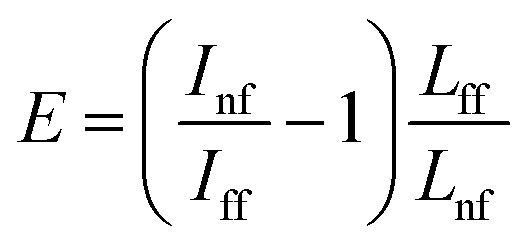


We took the tip radius ∼30 nm and laser focal radius ∼500 nm. The enhancement factor *E* ∼ 2.6 × 10^3^ was calculated using eqn (4) with the far and near field G bands' intensities shown as black and olive curves, respectively in the Fig. 4. Taking tip radius as the size of the near-field signal obviously underestimates the enhancement factor. Our analysis shows that spatial resolution is ∼9 nm (inset Fig. 4). Thus, more accurate estimates can be obtained assuming the near-field size to be ∼10 nm. However, we don't aim to provide detailed analysis of the enhancement factor in this manuscript, rather we want to demonstrate its high values even in the noncontact mode.

### 3.2 Graphene oxide on top of oxidized silicon wafer

**Fig. 5:**
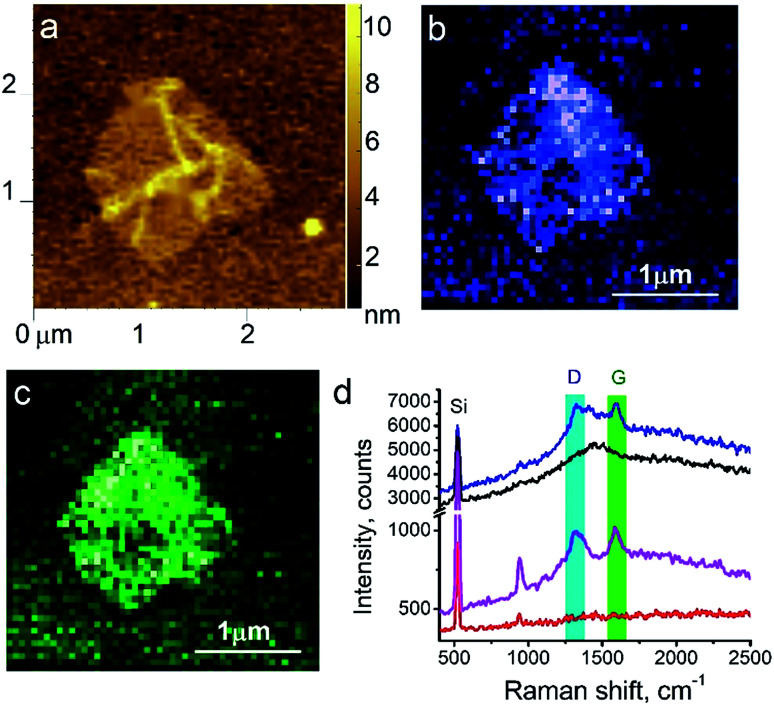
TERS of graphene oxide on silicon substrate. AFM topology (a); TERS mapping of D-band (b) and G-band (c) intensities; (d) TER spectra of GO (blue), oxidized Si wafer substrate (black), far-field Raman at tip withdrawn on 30 nm accumulated for 60 seconds (magenta) and 0.3 second (red).

The next material of interest was a graphene oxide (GO). GO was synthesized using a modified Hummers' method^8^ and deposited from an aqueous solution on piece of an oxidized silicon wafer. In this measurement, performance of a silver-coated tips in combination with 638 nm excitation wavelength of the laser at non-contact TERS experiment was tested. The laser power was set to ∼17 μW. The signal accumulation time was set to 0.3 s per point. The amplitude of tip oscillations was around ∼10 nm. The amplitude setpoint was set as 92% from free oscillations amplitude and negative phase. The Raman spectra of GO, similar to the spectra of carbon nanotubes, contain G and D bands. The Fig. 5 shows topology and Raman mapping of GO modes, clearly demonstrating a feasibility of non-contact TERS in chemical imaging. The TERS contrast (spatial resolution) was estimated to be about 50 nm, which is equal to the lateral distance between points in TERS and AFM measurements. The enhancement factor calculated using eqn (3) was *E* ∼ 187000 ≈ 2 × 10^5^.

### 3.3 Monolayer graphene on top of poly-(methyl methacrylate)

**Fig. 6:**
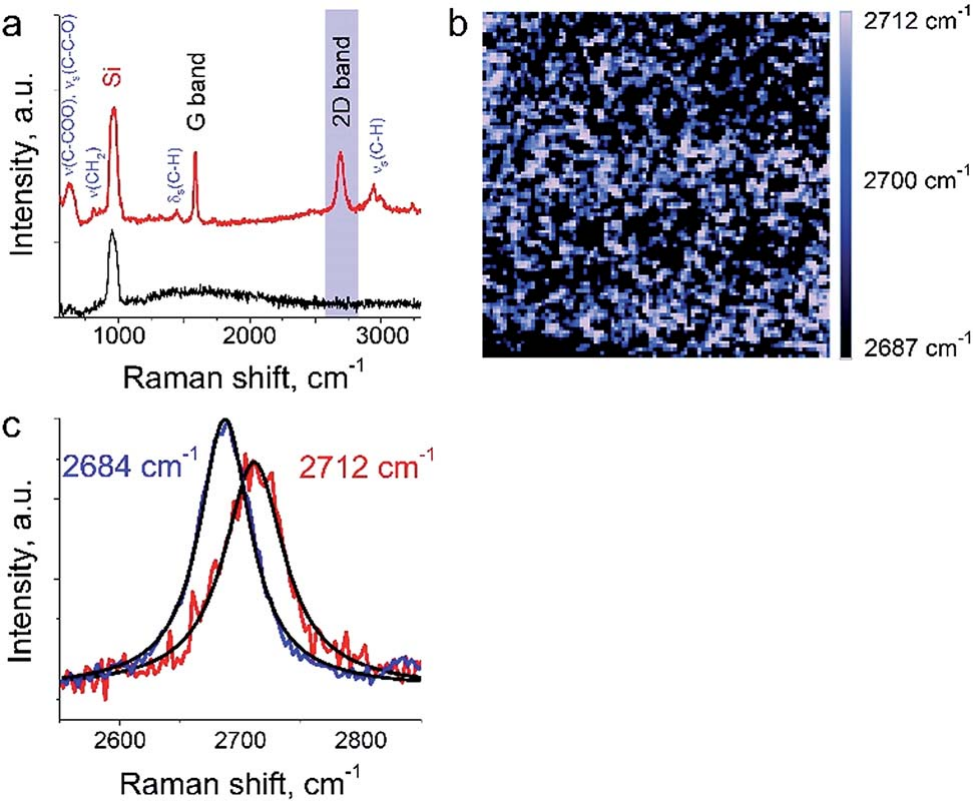
TERS of graphene on top of PMMA. (a) TER spectrum of graphene monolayer on PMMA when the tip is engaged (red curve) and withdrawn on a distance of 30 nm (black curve), (b) mapping of the 2D band intensity (image scale is 500 × 500 nm). (c) TER spectra at the tip positions corresponding to the highest (red) and lowest (blue) values of 2D band Raman frequency.

The aim of this work was to study a defect structure and distribution of strains in graphene during its transfer to silicone substrate using a very popular approach employing poly (methyl methacrylate) (PMMA) as a graphene support. It is assumed that the transfer and handling procedures introduce additional defects and stresses on graphene. Here we will present only a small part of the results since the details are beyond the primary scope of this manuscript. We show a mapping of 2D Raman mode frequency of graphene, which is used as a tracer of strain in carbon nanomaterials.^26^ This graphene/PMMA film is placed on a silicon wafer. The growth conditions, the crystal morphology and the transfer process of the graphene layer onto substrate are described elsewhere.^17^ The laser power used for the measurements was in the range from 12 to 25 μW. Exposure time was set to 0.5 s per spectrum (or point on TERS map). The amplitude of tip oscillations was ∼6 nm. The setpoint was set as 98% from free oscillations amplitude. During the first hour after the landing procedure, the measurements required several adjustments of the setpoint amplitude and phase, which preceded the TERS measurements. In the Fig. 6a the TERS spectrum of graphene on top of PMMA is shown when the tip is landed and withdrawn from the surface at a distance of 30 nm. Characteristic bands of graphene (G-band at 1585 cm^−1^, 2D band at ∼2700 cm^−1^) and PMMA (*ν*(C–COOH), *ν*_s_(C–C–O) at ∼620 cm^−1^, *ν*_s_(CH_2_) at 809 cm^−1^, *δ*_s_(C–H) at 1444 cm^−1^, and broad peaks at ∼3000 cm^−1^ assigned to *ν*_s_(C–H))^27,28^ were captured by TERS. A map of 2D band position shift is shown in the Fig. 6b. The 2D band in the Raman spectra of graphene is the most sensitive peak to stresses/deformation. According to Berciaud *et al.*^29^ the position of the band strongly depends on laser excitation, and for a 532 nm laser, should be located around 2670 cm^−1^. We found that the 2D band position varies from 2687 cm^−1^ to 2712 cm^−1^ with a total shift around ∼25 cm^−1^. These results suggest a nanoscale distribution of strong stresses in graphene prepared with this technology.

### 3.4 Mixture of a protein with drug

Here, we demonstrate TERS responses from a mixture of the Androgen Receptor (AR) activation function-1 domain (AF-1) protein^30^ and androgen receptor degrader (SARD) (UT-155) that binds selectively to the protein.

We aim at resolving the SARD binding sites in the protein where identification of SARD (drug) in the protein mixture and recognition of amino acids surrounding the SARD constitute the first step of our research. The mixture of SARD and AF-1 was prepared in a phosphate buffer. The molar ratio of component was set to 10 to 1 for the protein and SARD, respectively. The mixture was spin coated on a silicon wafer yielding a very thin layer of the composite. For this measurement, a 532 nm laser with power of 25 μW and acquisition time 0.5 s were used. The amplitude of tip oscillations was around ∼15 nm. The setpoint was set as 95% from free oscillations amplitude. Using eqn (2), the enhancement factor was estimated as *E* ≈ 3.6 × 10^4^.

**Fig. 7:**
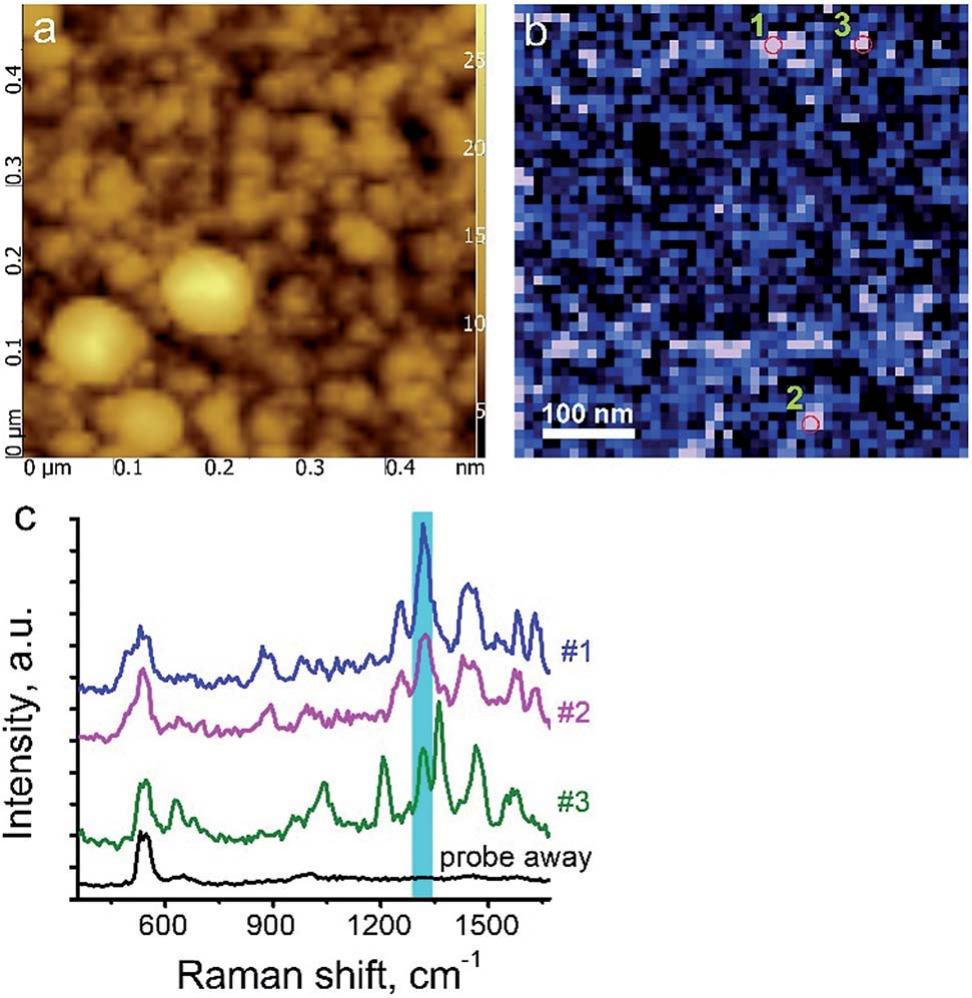
Protein–drug mixture. (a) AFM topology; (b) TERS mapping of intensity of Raman band at 1325 cm^−1^. (c) TER spectra acquired in positions shown in (b) with corresponding numbers and when the tip was withdrawn on 30 nm (black curve).

**Fig. 8:**
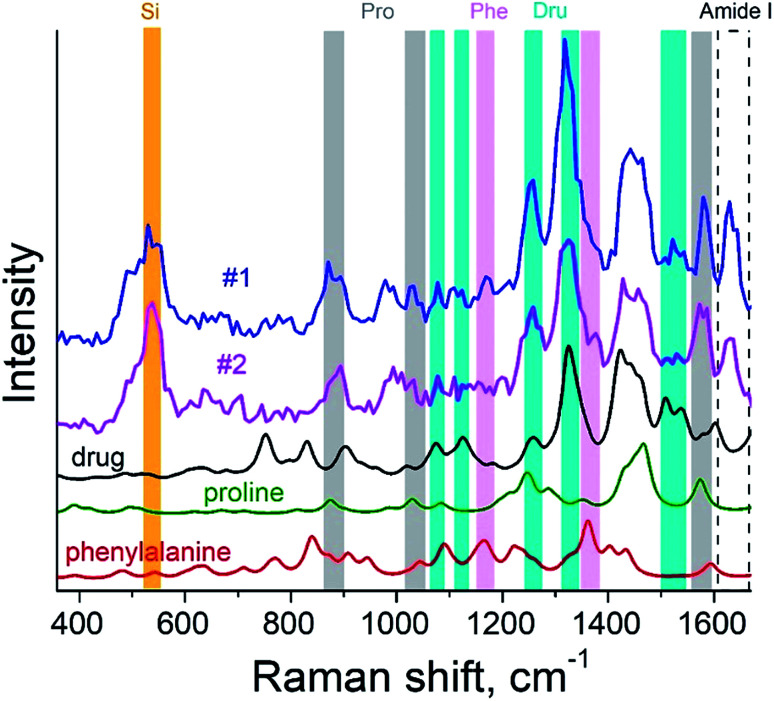
TER spectra of the protein–drug mixture (curves #1 and #2) and simulated Raman spectra of proline (green), phenylalanine (red) amino acids and drug (black).

The surface morphology and Raman mapping collected simultaneously are presented in Fig. 7a and b respectively, where the individual Raman spectra collected in different points marked in Fig. 7b are presented in Fig. 7c. Fig. 7b shows a distribution of the band at 1325 cm^−1^, marked by a blue rectangle in Fig. 7c and assigned to the presence of SARD. This peak assignment was made based on results obtained from DFTB^20,31^ calculations of Raman spectra for SARD and amino acids. Several selected Raman spectra representing the SARD and amino acids are shown as black, olive and red curves in the Fig. 8. We clearly see the signatures of the drug, amino acids, and amide I peak. According to literature, the amide I peak, located at around 1640 cm^−1^, contains information about the protein secondary structure.^32^ A presumable assignment of peaks in the experimental spectra is based on the simulation results and literature data and is shown in Fig. 8. The peaks at 1325 and 1500 cm^−1^ appear the least interfered by the peaks of different amino acids of the protein, so these peaks can be used to identify the drug in the mixture. We clearly see that the concentration of the drug depends on the position where the signal was collected. In position 1, the concentration of the drug is higher than in position 2. At this point we clearly see signatures of the proline and phenylalanine. While identification of all the amino acids is a challenging task, we are in the process of performing this task. We should note that our three attempts to measure TERS mapping of this mixture in a contact mode were completely unsuccessful due to severe tip contamination, regardless of setpoint and spring constant of cantilevers.

## 4. Conclusions

Here we demonstrated the capability of a TERS technique working in non-contact mode for study of various types of materials. Our study shows that non-contact TERS may provide significant enhancement of up to 10^5^. Although during measurements, in the non-contact mode the tip is far from the surface for about ∼80% of the time, the obtained TERS images of various materials clearly demonstrate feasibility of this technique. We believe that it can be more suitable than contact mode TERS for the study of materials with rough or sticky surfaces, biological objects, and for reducing probability of tip damage and contamination. It is notable that we were able to get below 10 nm spatial resolution during mapping of CNTs and strain distribution in graphene on top of PMMA.

## Conflicts of interest

There are no conflicts to declare.

## Supplementary Material

NA-001-C9NA00322C-s001
