# Supplementary material for: Noncontact tip-enhanced Raman spectroscopy for nanomaterials and biomedical applications
Source: Nanoscale Adv. 2019 Aug 19;1(9):3392–9. doi: 10.1039/c9na00322c (PMC9419720; doi:10.1039/c9na00322c)
Supplement: NA-001-C9NA00322C-s001 [file NA-001-C9NA00322C-s001.pdf]

This journal is © The Royal Society of Chemistry 2019

Electronic Supplementary Material (ESI) for Nanoscale Advances.

Noncontact tip-enhanced Raman spectroscopy for nanomaterials and biomedical applications

Dmitry N. Voylov [a,b*], Vera Bocharova [b], Nick Lavrik [c], Ivan Vlassiouk [d], Georgios Polizos [d], Alexei Volodin [e], Yuri M. Shulga [f], Alexander Kisliuk [b], Thirumagal Thiyagarajan [g], Duane D. Miller [h], Ramesh Narayanan [g], Bobby G. Sumpter [c] and Alexei P. Sokolov [b]

[a] *Department of Mechanical Engineering, Tufts University, Medford, Massachusetts, 02155, United States*

[b] *Chemical Sciences Division, Oak Ridge National Laboratory, Oak Ridge, Tennessee 37831, United States*

[c] *Center for Nanophase Materials Sciences and Computational Sciences and Engineering Division, Oak Ridge National Laboratory, Oak Ridge, Tennessee 37831, United States*

[d] *Energy & Transportation Science Division, Oak Ridge National Laboratory, Oak Ridge, Tennessee 37831, United States*

[e] *Institute of Problems of Chemical Physics RAS, Chernogolovka, Moscow region, 142432, Russia*

[f] *National University of Science and Technology MISIS, Moscow, 119049, Russia*

[g] *Department of Medicine, University of Tennessee Health Science Center, Memphis, Tennessee 38103, United States*

[h] *Department of Pharmaceutical Sciences, University of Tennessee Health Science Center, Memphis, Tennessee 38103, United States*

Experimental Details

During this work 15 regular "as received" and 32 oxidized probes were tested for enhancement. The enhancement was tested using side illumination configuration with 100x objective with two types of samples: Graphene oxide on Si substrate and polycrystalline Silicon. Polycrystalline Silicon was used primarily because of less tendency for contamination with comparably low roughness of the surface. Tips were considered to exhibit a good TERS activity if a Raman peak intensity of near-field signal was at least 5 times higher than that at a far-field. In other words, to be considered as a TERS active, each tip should enhance a Raman peak intensity for at least 5 times when it approached the surface in compare with micro Raman signal with the tip was withdrawn for 30-50 nm from the surface. In the Fig. S1 optical images of some as received and oxidized probes exhibited Raman enhancement are shown. According to our statistics about 88% of oxidized and only ~66% of non-oxidized showed enhancement of the Raman signal (28 out of 32 of oxidized and 10 out of 15 of "as received" probes respectively). This statistic excludes the probes with excessive thickness or lack of metal on the tip (Fig. S2).

Based on our statistics we estimated a shelf-life time of the TERS probes as 60 days in average. Six out of ten TERS probes exhibited Raman enhancement after 60 days of storing in ambient environment.

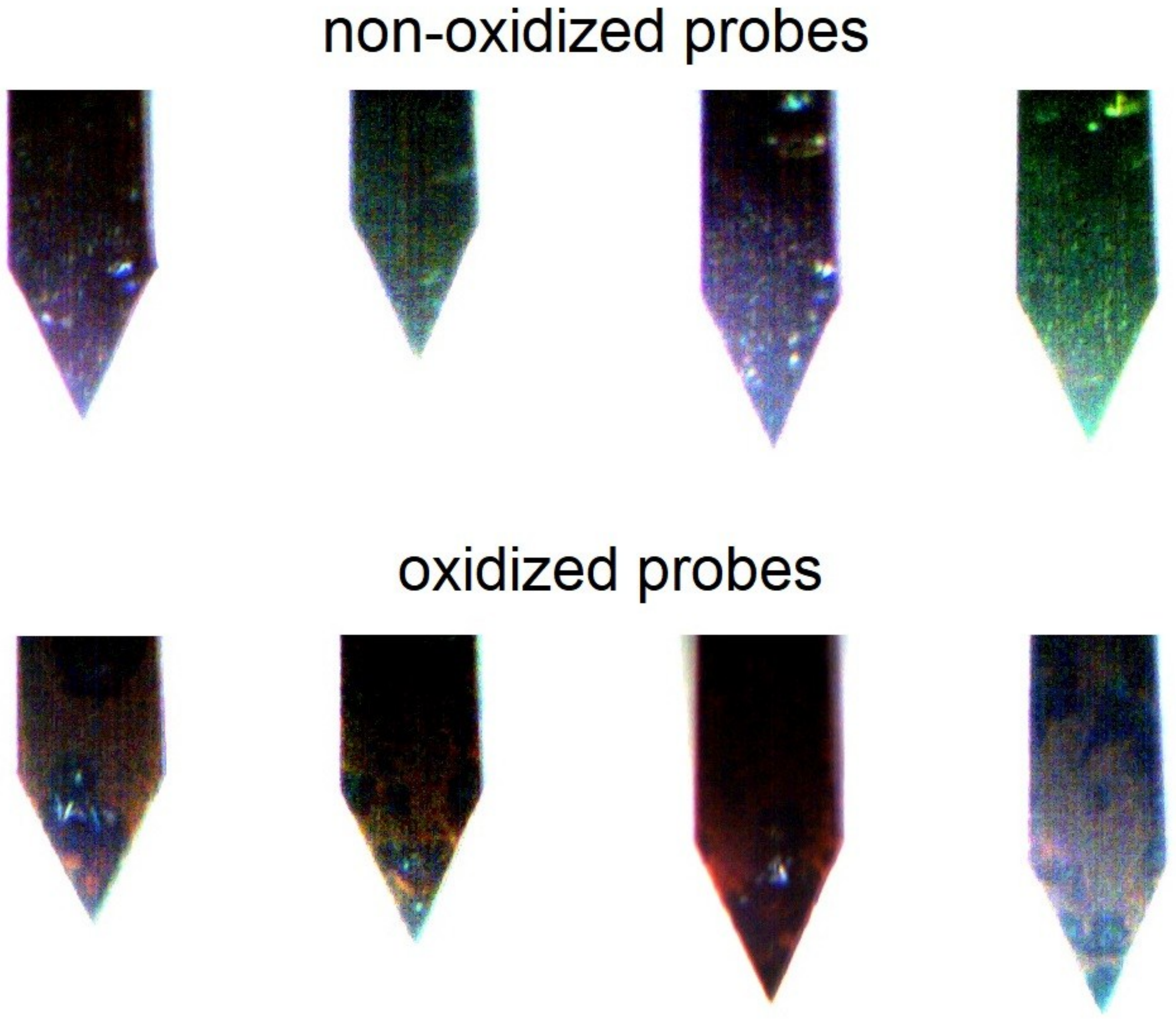

Figure S1. Optical images of “as received” non-oxidized (top) and preliminarily oxidized (bottom) probes after metallization obtained from the top using 10x objective.

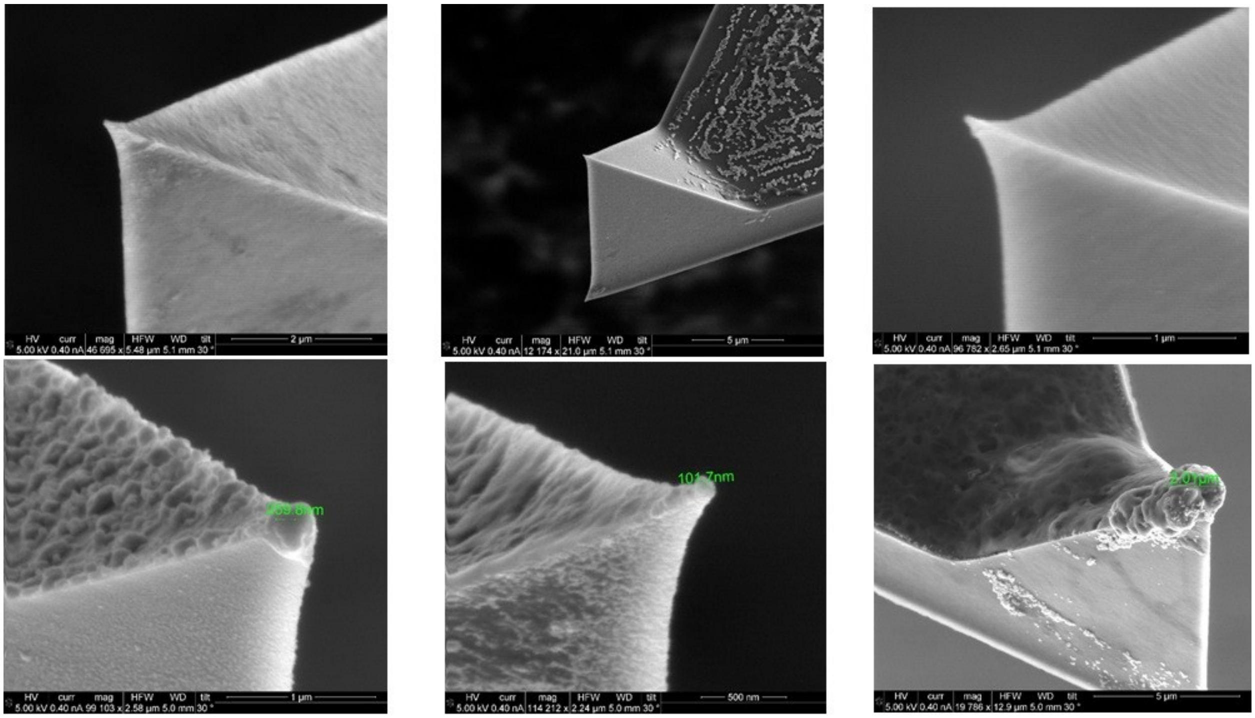

Figure S2. SEM images of metallized probes showed Raman enhancement (top 3 images) and no enhancement (bottom 3 images).

Figure 3 represents optical images from a side view of the probes before (left image) and after manual laser alignment.

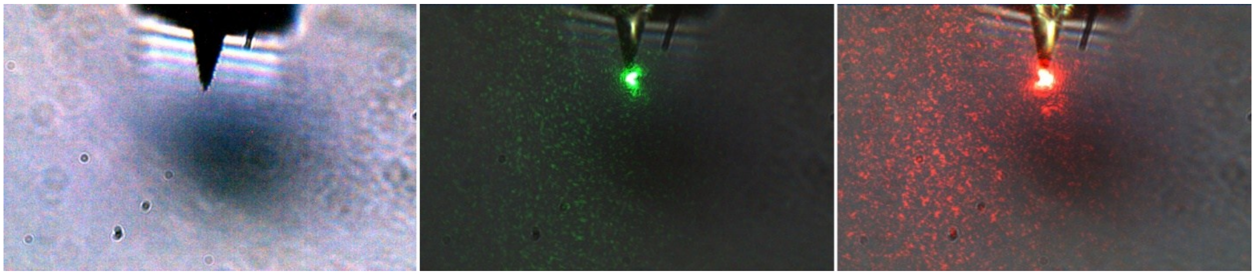

Figure S3. Optical image of different probes before and after manual alignment of 532 and 638 nm lasers obtained from the side using 100x objective.

Noncontact regime.

All TERS measurements were done using noncontact AFM mode, means that the resulting force between tip and surface was attractive. In order to maintain non-contact regime, the feedback system was set to control both amplitude and negative phase shift setpoints. During our tests we didn’t find any benefits from using frequency shift as a control parameter of noncontact regime, which we believe is because of all our measurements were done in ambient conditions (without vacuum). The cantilever oscillation amplitude was set in the range from 92% to 97% of free amplitude (when the tip is retracted (withdrawn) from the surface) and adjusted afterwards. Estimated oscillation amplitude was in a range between 5-15 nm. The choice of setpoint amplitude and phase was based only on stability and quality of resulting AFM

images and TERS spectra which in turn depended on surface properties and roughness of materials. Besides typical AFM alignment and tip engagement, the TERS measurement required preliminary adjustments due to temperature fluctuations and sample drift. It was crucial to let the system equilibrate after turning electronics on for at least one hour before AFM and Raman alignments. However, regardless of this equilibration, tip amplitude and phase required additional adjustments before TERS experiments due to significant deviations in time.
